# Prematurity Appears to Be the Main Factor for Transient Congenital Hypothyroidism in Greece, a Recently Iodine-Replete Country

**DOI:** 10.3390/nu17193039

**Published:** 2025-09-24

**Authors:** Eftychia G. Koukkou, Panagiotis Girginoudis, Michaela Nikolaou, Anna Taliou, Alexandra Tsigri, Danae Barlampa, Marianna Panagiotidou, Ioannis Ilias, Christina Kanaka-Gantenbein, Kostas B. Markou

**Affiliations:** 1 Department of Endocrinology, Diabetes and Metabolism, “Helena Venizelou” Maternity Hospital, 11521 Athens, Greece; 2 Institute of Child Health, Agia Sophia Children’s Hospital, 11527 Athens, Greece; 3 Unit of Endocrinology, Diabetes and Metabolism, First Department of Pediatrics, Medical School, Agia Sophia Children’s Hospital, National and Kapodistrian University of Athens, 11527 Athens, Greece; 4 Department of Endocrinology, Hippokration General Hospital, 11527 Athens, Greece; 5 Department of Internal Medicine, Division of Endocrinology, University of Patras, 26504 Patras, Greece

**Keywords:** iodine intake, Transient Congenital Hypothyroidism, congenital hypothyroidism, iodine deficiency, prematurity

## Abstract

**Background/Objectives**: Neonatal screening programmes for thyroid function testing, based on thyroid-stimulating hormone (TSH) assessment, detect both Permanent Congenital Hypothyroidism (PCH) and Transient Congenital Hypothyroidism (TCH). Maternal iodine-deficient dietary intake may result in compensatory neonatal TSH elevation; screening for Congenital Hypothyroidism (CH) is used as an indicator of the degree of iodine deficiency and of its control. In Greece, newborn screening for CH, using TSH measurement in dried blood spots (Guthrie card), began in 1979 through the Institute of Child Health (ICH). Although the general Greek population is considered iodine-replete, most pregnant Greek people are mildly iodine deficient according to the stricter WHO criteria. The aim of this retrospective study was to record the cases of TCH and the main causative factors over a 10-year period (2010–2019) in Greece, when the country was deemed to be iodine-replete. **Methods**: The number of births in Greece between 2010 and 2019 was retrieved from the Hellenic Statistical Authority (ELSTAT) archives: 952,109 births were recorded. The total number of newborns assessed through the ICH was 951,342 (99%). During this period, 22,391 newborns were identified to have TSH > 7 mIU/L after the second check on the initial card. Among those, 17,992 underwent retesting with a serum sample. Out of the retested newborns, 1979 were screened positive for CH and immediately began treatment with levothyroxine. We followed up with families, paediatricians, and paediatric endocrinologists to determine whether L-thyroxine therapy had been successfully discontinued for at least two months after the child’s third birthday. Successful contact was achieved with 889 individuals. From this group, 329 children had successfully discontinued thyroxine, classified as TCH. Demographic data, including gender, gestational age, and birth weight, were collected from the archives of the ICH. Maternal data, including thyroid medication use and the presence of elevated thyroid autoantibodies during pregnancy and childbirth, were also recorded. **Results**: Logistic regression analysis revealed that, while controlling for all other predictor variables, the odds ratio of transient hypothyroidism was 2.078 (95% CI: 1.530 to 2.821) for prematurely born children compared to those born at term. The effects of other factors on TCH versus PCH were not significant. **Conclusions**: It seems that prematurity is the main factor contributing to Transient Congenital Hypothyroidism in Greece, a recently iodine-replete country.

## 1. Introduction

Neonatal thyroid function testing, primarily based on thyroid-stimulating hormone (TSH) assessment, can be used to detect not only Permanent Congenital Hypothyroidism (PCH), which has an incidence of approximately 1 in 4000 births, but also Transient Congenital Hypothyroidism (TCH) [1]. The incidence of TCH varies and can be as high as 1 in 10 newborns, with the main cause being iodine deficiency (ID). Robust evidence supports a cause-and-effect relationship between iodine deficiency and the pathogenesis of TCH [2,3,4]. In affected children, normal thyroid function is typically achieved by the end of the third year of life, allowing discontinuation of levothyroxine treatment. Normal thyroid function is subsequently maintained into adulthood, similar to other healthy individuals [5,6]. Maternal iodine-deficient dietary intake may result in compensatory neonatal TSH elevation and screening for Congenital Hypothyroidism (CH) is used as an indicator of the degree of ID and of its control.

Following the introduction of newborn screening for Congenital Hypothyroidism, the incidence of TCH in Europe, where iodine deficiency has historically been prevalent, was reported to be nearly eight times higher than in North America, where iodine deficiency was addressed decades earlier [7]. The role of iodine deficiency in the aetiology of TCH has also been confirmed through studies demonstrating the preventive effects of iodine supplementation [8].

In Greece, newborn screening for Congenital Hypothyroidism (CH) using TSH measurement in dried blood spots (Guthrie Card) began in 1979 through the Institute of Child Health (ICH). The first dataset, collected one year after the program’s initiation from a sample of 75,879 newborns, reported a prevalence of CH (including both permanent and transient forms) of 1 in 4200 [9]. A subsequent report in 1994, based on a larger sample of 1,274,000 newborns, showed a prevalence of CH at 1 in 3379. Notably, in this report, the TSH cut-off for the initial screening was set at 30 mU/L. In the same study, “false positive” cases were reported at a prevalence of 1 in 368, amounting to 3459 cases. However, the term “false positive” was used differently to how it is now used in our current understanding of TCH [10]. This term was used when a TSH value over 30 mU/L was found only in the first measurement of initial card and was not confirmed in the repeated analysis in another blood spot from the initial card. Currently, TCH refers to CH which spontaneously resolves in the first few months or years of life and the diagnosis is established following the withdrawal of levothyroxine therapy around 3 years of age [5]. The estimated overall prevalence of TCH, irrespective of causative factors, was 1 in 14,154 [11]. From the year 2012, the cut-off point was further reduced to 7 mU/L.

Although the general Greek population is now considered to be iodine-replete, the majority of pregnant Greek people are considered mildly iodine-deficient according to higher cut-off values of Urinary Iodine Excretion in the WHO criteria, with a median Urinary Iodine Excretion (UIE) of 127.1 μg/L [12]. During pregnancy, iodine requirements are increased due to both the normal increase in maternal thyroid hormone production and the transfer of iodine to the foetus for embryonic thyroid hormone production from approximately the 17th week onwards. Insufficient iodine intake during this period has negative effects on maternal thyroid homeostasis, as expressed by an increased TSH [13]. A clinical study conducted in Athens in 2012, which monitored iodine intake in a sample of pregnant people during their first trimester, found that more than 50% of participants had UIE levels below 100 µg/L, and one-third of participants had levels below 50 µg/L, indicative of mild and moderate iodine deficiency, respectively [14]. Despite this, the entire population of Greece has adequate dietary intake of iodine.

The aim of our retrospective study was to record the cases of TCH and the main causative factor over a 10-year period (2010–2019) in Greece, a period when the country was iodine-replete. We sought to analyse the factors contributing to the occurrence of TCH and study its specific aetiopathogenic characteristics in conjunction with existing data on daily iodine intake during the same period.

## 2. Materials and Methods

### 2.1. Data Collection

Data collection for this retrospective study took place in 2025. The number of births in Greece between 2010 and 2019 (Group A) was collected from the Hellenic Statistical Authority (ELSTAT) archives [15]. Additionally, from the archives of the Institute of Child Health (ICH) in Greece, which covers newborn screening across the country, we recorded all newborns screened for CH during the same period (Group A1). TSH levels were measured in dried blood spots using Guthrie cards. Blood samples were collected via heel prick between the third and fifth day of life, typically prior to hospital discharge, and the cards were mailed to the ICH daily or every second day.

If a TSH value on the Guthrie card exceeded 7 mU/L, a second measurement was performed in duplicate using the initial card. TSH levels below 7 mU/L on the repeat specimen were considered normal, and no further action was undertaken, as the infant was typically over 1 month old at the time of re-examination. A TSH value above 7 mU/L on the initial cards after the repeated test was considered positive for CH, and the newborn was referred for biochemical (serum TSH and FT4 levels) and clinical evaluation. CH was confirmed, and treatment with L-thyroxine was initiated if serum TSH levels exceeded 10 mU/L.

For all identified cases of CH, we followed-up with families, paediatricians, and paediatric endocrinologists to determine whether L-thyroxine therapy had been successfully discontinued for at least two months after the child’s third birthday. Demographic data, including those on gender, gestational age, and birth weight, were collected from the archives of the ICH. Maternal data, including thyroid medication use and the presence of elevated thyroid autoantibodies (antithyroglobulin or antiperoxidase) during pregnancy and childbirth, were also recorded (Group B). Newborns were categorized as full-term (Group B1) or premature (Group B2). From this group of identified CH cases with successful contact with families and doctors, we classified cases that had successfully discontinued thyroxine treatment as presenting with TCH (Group C). The remaining cases were considered to have PCH (Group D).

**Figure 1:**
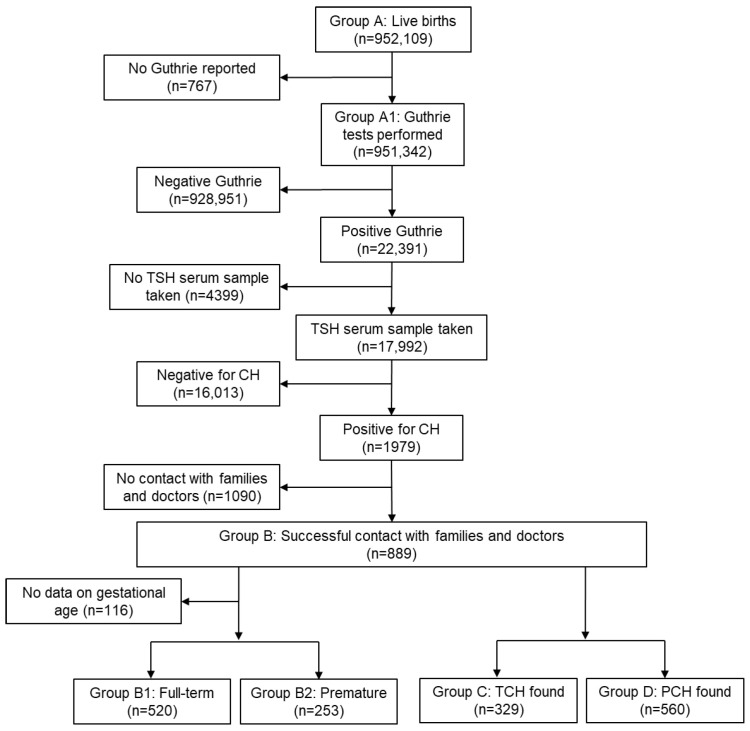
A flowchart of data collection and preliminary analysis.

A flowchart of the data collection and preliminary analysis processes is shown in Figure 1.

### 2.2. Data Selection

To identify the TCH cases not influenced by maternal factors during gestation, the following subgroups were subsequently defined:

Group C: Children who successfully discontinued thyroxine therapy, classified as TCH.

Group D: Children who continued thyroxine therapy, classified as PCH.

- Group C1/D1: Full-term babies.
- Group C2/D2: Premature babies.
- Group Ca:/Da: Children whose mothers had elevated thyroid autoantibodies but normal thyroid function tests (TFTs), and not receiving thyroid medication during pregnancy.
- Group C3:/D3: Children whose mothers received thyroid medication during pregnancy.
  a Group C3a/D3a: Full-term babies.
  b Group C3b/D3b: Premature babies.

**Figure 2:**
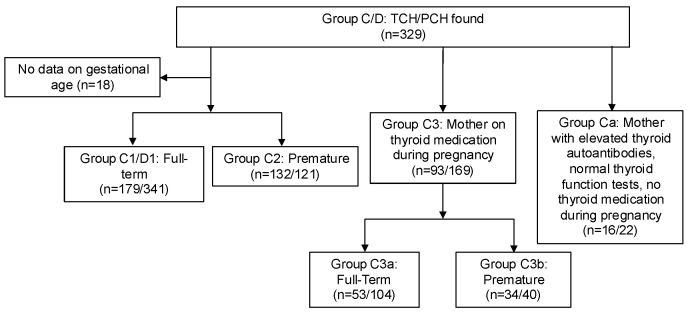
A flowchart of the selection of Transient and Permanent Congenital Hypothyroidism (TCH, PCH) cases included in the analysis.

To identify TCH cases not influenced by maternal factors during gestation, such as medication use or elevated thyroid autoantibodies, we excluded Groups Ca and C3 from the total of Groups C1 and C2, resulting in the Target Group of our study. This group was further divided into full-term (Target Group 1) and premature (Target Group 2) infants. A flowchart of the selection of the cases included in the analysis is shown in Figure 2.

### 2.3. Laboratory Measurements

Commercially available reagents from Roche Diagnostics (Mannheim, Germany) were used to measure serum TSH, antithyroglobulin antibodies, and antiperoxidase antibodies. Analyses were performed on an Elecsys 2010 apparatus (Hitachi, Tokyo, Japan) using an electrochemiluminescence technique [16].

### 2.4. Statistical Analysis

Statistical analyses were performed using SPSS statistical software (version 17.0; Chicago, IL, USA), following the appropriate methodological approach [17]. Statistical significance was defined as *p* < 0.05. Where appropriate, Student’s *t*-test was used to compare the means of two numerical variables, while Pearson’s χ^2^ test was employed for comparisons of proportions. Logistic regression analysis was conducted to examine the relationship between the centile of birth weight, premature birth (1 = yes, 0 = no), sex of the child (female = 1, male = 0), maternal treatment for hyper- or hypothyroidism (1 = yes, 0 = no), and the presence of increased maternal antithyroid antibodies with normal thyroid function (1 = yes, 0 = no) with the occurrence of transient hypothyroidism in children (1 = yes, 0 = no).

### 2.5. Ethical Considerations

The study received approval from the local ethics committee of the Institute of Child Health (Decision Number 740A/16.7.2025). Informed consent was obtained from the parents or legal guardians of the participating children.

## 3. Results

The total number of births in the decade 2010–2019 according to the ELSTAT data was 952,109.

The total number of newborns assessed in the period 2010–2019 in the ICH in Greece was 951,342 (99%) (Group A1). During this period, 22,391 newborns were detected with TSH > 8 mIU/L after the second check on the initial card. Among those, 17,992 underwent retesting with a serum sample, while 4399 cases could not be re-assessed. Out of the retested newborns, 1979 were found to be positive for CH and immediately began treatment with levothyroxine.

**Figure 3:**
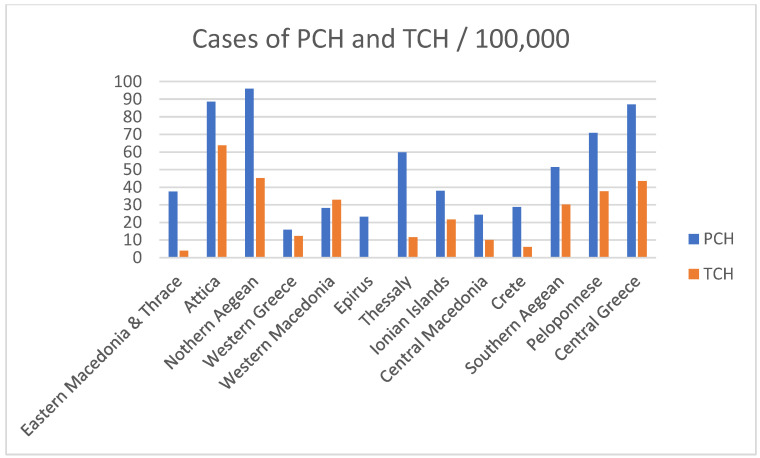
The prevalence of Permanent Congenital Hypothyroidism (PCH) and Transient Congenital Hypothyroidism (TCH) per 100,000 children in the geographic regions of Greece as recorded in 889 individuals after successful contact, who screened positive for Congenital Hypothyroidism and immediately began treatment with levothyroxine.

After contacting the families and doctors of these cases, successful contact was made with 889 individuals (Group B). From this group, it was found that 329 children had successfully discontinued thyroxine treatment (Group C). These cases are classified as presenting with TCH. The remaining 560 cases were considered to have PCH (Group D). Figure 3 presents prevalence of Group B per 100,000 children in the geographic regions of Greece.

The results for 300 cases were available regarding maternal thyroid autoimmunity positivity with normal thyroid function (Groups Ca and Da) and maternal thyroid treatment (Groups C3 and D3). Further results are shown in Table 1 and Table 2.

The logistic regression analysis revealed that, while controlling for all other predictor variables, the odds ratio of transient hypothyroidism was 2.078 times (95% CI: 1.530 to 2.821, *p* = 0.001) in prematurely born children compared to those born at term. The effect of other predictors on transient versus permanent hypothyroidism was not significant.

The cases not influenced by maternal factors during gestation, such as medication use or elevated thyroid autoantibodies were 262 (female children = 140; male children = 122). Of these, 95 presented with TCH (Target Group) while the rest were classified as PCH. The only determining factor for PCH is sex: female children had an odds ratio of 1.86 (1.06–3.26, *p* = 0.029).

## 4. Discussion

The primary objective of this retrospective study was to document cases of TCH in Greece from 2010 to 2019 and analyse contributing factors. Data from this decade suggest that Greece was iodine-replete, except for sporadic cases of iodine deficiency among pregnant people as defined in stricter WHO criteria [12,18].

Our stepwise approach to analysing TCH cases identified key factors influencing its occurrence, including prematurity, maternal autoimmune thyroid disease, and maternal use of medications affecting thyroid function. Genetic causes of TCH, such as mutations in factors influencing thyroid hormone production, are rare, and goitrogens are not a dietary concern for Greek pregnant people [13]. Iodine availability remains the most significant exogenous factor influencing thyroid hormone biosynthesis and TCH occurrence [5].

Prematurity emerged as the leading cause of TCH in our study. This finding persisted across the total cohort and subgroups, including those exposed to adverse maternal environments (e.g., increased thyroid autoantibodies or maternal thyroid medications) and those without such influences. It is known that the risk for TCH increases with the degree of prematurity [19,20]. There is robust evidence supporting a causal relationship between inadequate iodine intake and TCH [1,2,21]. Studies have also demonstrated that iodine supplementation prevents TCH [8]. Prematurity is one of the most important factors for TCH development, especially in iodine-sufficient countries. Other causes of TCH are maternal exposure to thyroid medications, increased maternal thyroid autoantibodies, the use of iodine-based skin disinfectants on premature infants or, of course, untreated maternal hypothyroidism [22]. Taking the above into consideration, in our analysis, we excluded all the cases with the above-mentioned parameters that contribute to TCH in newborns, with the exception of the use of iodine-based skin disinfectants used on the umbilical cord remnants of newborns or prior to umbilical line placements, since no relevant records exist and such products are widely available in Greece. Public maternity hospitals do not use topical iodine-containing disinfection agents during childbirth. However, we do not have concrete information about the use of such agents in private maternity hospitals; therefore, we cannot exclude that some cases of TCH in our study are related to the use of topical iodine-containing disinfection agents. The incidence of TCH increases with the lower gestational age and is attributed to immaturity of the thyroid function to respond to a variety of factors, such as those mentioned above [20]. Our findings are in line with the existing literature data, both regarding the total number of TCH identified as well as in the Target Group.

In agreement with previous reports, we found interaction between prematurity/permanent hypothyroidism and birthweight centile. This finding indicates different maturity levels of the hypothalamic–pituitary–thyroid axis in small-for-gestational-age babies [22,23]. This may be attributed to genetic factors, maternal nutritional status, placental function and transfer of nutrients, intrauterine hormones, and growth factors [24]. In addition, the potential for collinearity between prematurity and birth weight centile cannot be fully excluded. However, we did not perform a mediation analysis to further explore the relationship between prematurity and birth weight centile, as our sample size was insufficient to support a robust and meaningful analysis [25].

The prevalence of TCH varies globally, probably due to differences in iodine intake. For example, TCH accounts for 5–65% of diagnosed Congenital Hypothyroidism (CH) cases worldwide. In North America, where iodine sufficiency has been established for many years, TCH prevalence is approximately 10% [26,27]. Neonatal thyroid is particularly susceptible to iodine deficiency due to its low iodine content at birth and higher iodine turnover compared to adults [1]. Increased iodine requirements during the neonatal period may prevent some neonates with underlying vulnerabilities from developing normal thyroid function, necessitating levothyroxine treatment. A subset of these children eventually regains normal thyroid function and discontinue treatment [6]. Indicators of TCH include lower levothyroxine requirements during treatment, lower TSH levels at diagnosis, a normally positioned thyroid gland, lower screening TSH levels, higher FT4 levels at diagnosis, low birthweight, male sex, and the absence of TSH elevation during treatment [28,29]. In addition, the recorded prevalence depends on the cut-off TSH value used and the optimal cut-off value remains a topic of debate [30,31].

The newborn screening program, initiated in Greece in September 1979 and performed in the ICH, has significantly improved child health. The most recent study in 2010 estimated the overall prevalence of TCH as 1:14,154, including cases from all causative factors [11]. The commonest cause of CH in Greece is ectopy of the thyroid gland followed by aplasia of the thyroid [32]. In the current study, we excluded TCH cases influenced by exogenous maternal factors (e.g., maternal thyroid disease or goitrogens) to identify a “Target Group” with TCH attributable solely to insufficient iodine intake. The observed prevalence of TCH in relation to the total number of detected cases with CH is 1:6. Meanwhile, the noted incidence of TCH in relation to births is 1:3061. For the Target Group of our study, we found an incidence of TCH in relation to the total detected cases with CH to be 1:21 and the incidence in relation to births at 1:10,000. The prevalence and incidence rates of TCH in this group therefore reflect the sufficient iodine intake in Greece.

Maternal antibodies and maternal treatment were not significant predictors, despite being considered relevant factors. Our findings are in accordance with longitudinal studies suggesting that these patients exhibit minor congenital thyroid function abnormalities requiring long-term follow-up [33].

Regarding sex, the only determining factor is female sex for PCH. This finding is in accordance with previous reports [34]. The preponderance of female cases is mostly associated with dysgenesis of the thyroid gland [32,35]. Female sex is associated with an increased overall risk of CH, but this trend is less pronounced in TCH compared to PCH. While studies show a higher female to male ratio in total CH cases, likely due to an increased incidence of thyroid dysgenesis in females, the specific relationship between female sex and TCH is less clear and can vary. These discrepancies may be attributed to different reasons such as heterogeneity in the sample size, study design, different prevalence across the populations, and differing statistical methods [36,37].

Despite improvements in iodine status across Europe, including the Balkan Peninsula, ID remains a concern in some regions. In Greece, efforts to address ID began approximately 50 years ago, when iodopenic goiter was still endemic [38,39]. Earlier studies reported urine UIE ranging from 20 to 50 µg/L, indicating moderate–severe ID. Gradual improvements have been achieved through iodized salt use, better transportation, and higher living standards. Recent reports suggest that Greece is now iodine-replete, though mild ID persists in some areas [18,40]. A study conducted in Athens in 2012 reported that over 50% of the pregnant people participating had UIC < 100 µg/L, with one-third having UIC < 50 µg/L, indicating mild–moderate ID [13]. A nationwide survey in 2018 found a median UIC of 127.1 µg/L, confirming adequate iodine intake overall [12]. The scattered distribution of TCH cases in our Target Group aligns with these findings, suggesting local variations in iodine intake. We cannot rule out that some cases of TCH are due to insufficient maternal iodine intake.

We strongly support the suggestion by the 2020–2021 updated European CH consensus guideline that TSH levels are retested at the second postnatal week of life or 2 weeks after the first screening in preterm and low-birth-weight infants [41,42].

Our study’s main strength is the identification of TCH cases solely attributable to insufficient iodine intake, separated from other causative factors. This distinction allows for a detailed comparison of characteristics and contributing factors. However, limitations include the retrospective design and missed cases due to challenges in follow-up, particularly among transient populations, especially immigration flows from Asia and residents of remote areas. We acknowledge that the high rate of lost-to-follow-up participants is a significant limitation in our study. This low rate of follow-up is mostly a result of the inability to obtain up-to-date contact details. We consider that a significant number of these lost-to-follow-up cases correspond to transient populations migrating to Northern and Western Europe and, in particular, immigration flows from Asia to Greece. During the time period studied, Greece was the host country to great numbers of immigrant people who were of reproductive age and whose stay in the country was temporary. Consequently, we believe that our sample reflects the status of Greece on the subject we studied.

Future efforts should focus on addressing iodine deficiency in vulnerable populations and improving access to healthcare services for comprehensive newborn screening, especially in Southeastern Europe [43].

## 5. Conclusions

Our findings support the theory that prematurity is the main factor for Transient Congenital Hypothyroidism in Greece, a recently iodine-replete country. The increased global prevalence of CH since 1979, particularly in regions like the Eastern Mediterranean area, highlights the importance of improved detection through lower screening thresholds and advances in screening methods. Factors contributing to this increase include higher preterm birth rates and improved neonatal survival. They also underscore the need for robust follow-up programs and the development of guidelines for the management of CH.

## Figures and Tables

**Table 1:** Sex distribution by group; * data for fewer subjects than the entire Groups C or D.

Group	Male Children (*n*)	Female Children (*n*)
C1—term birth	103	76
C2—premature birth	62	70
Ca *, maternal increased thyroid antibodies	16	25
Ca′ *, maternal normal thyroid antibodies	82	66
C3 *, birthing parent on Rx/No Rx	51/102	42/81
D1—term birth	154	187
D2—premature birth	61	60
Da *, maternal increased thyroid antibodies	30	39
Da′ *, maternal normal thyroid antibodies	141	161
D3 *, birthing parent on Rx/No Rx	82/157	87/184

Rx: thyroid medication during pregnancy.

**Table 2:** Distribution of newborns according to their gestational age and their classification for having Transient Congenital Hypothyroidism (TCH) or Permanent Congenital Hypothyroidism (PCH).

Hypothyroidism	Birth	*n*	Gestational Age (Weeks) Mean ± SE
Transient	Term	179	36.31 ± 2.07
Permanent	Term	341	40.88 ± 1.51
Transient	Premature	132	29.74 ± 2.48
Permanent	Premature	121	35.16 ± 2.55

## Data Availability

The original contributions presented in this study are included in the article. Further inquiries can be directed to the corresponding author.
